# A new mindfulness and psycho-educative program for treatment of brain fatigue, evaluated after an acquired brain injury and multiple sclerosis

**DOI:** 10.1080/21642850.2025.2502039

**Published:** 2025-05-07

**Authors:** Gustaf Glavå, Lars Rönnbäck, Birgitta Johansson

**Affiliations:** a Department of Psychology, University of Gothenburg, Gothenburg, Sweden; b Institute of Neuroscience and Physiology, Department of Clinical Neuroscience, University of Gothenburg, Gothenburg, Sweden

**Keywords:** Fatigue, neurorehabilitation, acquired brain injury, multiple sclerosis, mindfulness

## Abstract

**Background:** Fatigue is a common long-term problem after illnesses affecting the brain, having substantial impact on work ability, social activities, and quality of life. Treatment has been requested in the healthcare and by patients. The aim of this randomized pilot study is to evaluate a new Brain Fatigue and Mindfulness program (BF-M) for participants suffering from long-term fatigue after an acquired brain injury or Multiple Sclerosis. BF-M consists of six biweekly group meetings. Meditation is practiced, knowledge is imparted about fatigue and participants have time to share and discuss common issues.

**Methods:** A mixed method research design is used with quantitative and qualitative methods. Sixteen participants completed BF-M and 16 controls on waitlist responded to questionnaires before and after the intervention.

**Results:** After the program, the BF-M group had a significant reduction in BF and anxiety compared to the controls. The analysis of 13 interviews with BF-M participants suggested that: (1) BF-M became a space for the participants to exchange and share experiences associated with BF; (2) the participants felt more understood and less alone; and (3) they learned how to better understand and live with brain fatigue.

**Conclusions:** This study showed that BF-M may reduce brain fatigue and help participants to better manage their difficulties. Based on this pilot study, we suggest that BF-M may be considered as a rehabilitation option for brain fatigue. However, more confirmatory research with larger and different patient groups is needed.

## Introduction

Fatigue is a significant common long-term problem after many illnesses affecting the brain, e.g. after stroke, traumatic brain injury (TBI), brain tumor, brain inflammation, neurological diseases such as Multiple Sclerosis (MS) and also indirectly after cardiac arrest with limited or no blood flow to the brain (Cantor et al., 2008; Diaz-Arias et al., 2021; Hagell & Brundin, 2009; Joshi et al., 2023; Kluger et al., 2013; Shah, 2009; Staub & Bogousslavsky, 2001). The prevalence of fatigue is high, although the figures vary depending on the assessment methods used. After a stroke fatigue is estimated to occur in between 26% and 77% of patients, for TBI between 45% and 73%, for MS between 38% and 83% (Kluger et al., 2013) and one-third suffer from long-term fatigue after cardiac arrest (Joshi et al., 2023). Fatigue is regarded as a primary symptom distinguishable from depression (Cantor et al., 2008; Johansson & Rönnbäck, 2014a; Kluger et al., 2013). Although depression is common and can co-exist with fatigue, it is necessary to differentiate between the fatigue and the depression. Fatigue is reported as one of the most troublesome symptoms among patients (Pollock et al., 2014), resulting in difficulty taking part in everyday activities with reduced quality of life (Cantor et al., 2008; Glader et al., 2002; Hawthorne et al., 2009; van de Port et al., 2007) as well as reduced work performance (Andersen et al., 2012; Johansson, 2022).

Only a few treatment studies for fatigue after an acquired brain injury or MS are reported. Pharmacological studies with methylphenidate have reported reduced fatigue (Johansson, Carlsson, et al., 2012; Johansson, Wentzel, et al., 2015), or improved wakefulness (Kaiser et al., 2010; Stankoff et al., 2005). Additionally, non-pharmacological studies have reported reduced fatigue as a result of mindfulness (Acabchuk et al., 2021; Grossman et al., 2010; Johansson, Bjuhr, et al., 2012), cognitive behavioral therapy (CBT) (Nguyen et al., 2017), light therapy (Sinclair et al., 2014), and fatigue management interventions (Van Heest et al., 2017). For Chronic Fatigue Syndrome (CFS), graded exercise therapy (Moss-Morris et al., 2005), CBT (Kuut et al., 2024), and mindfulness (O'Dowd & Griffith, 2022) have shown benefits. However, review reports indicate positive results for methylphenidate (Ali et al., 2022), amantadine (Yang et al., 2017), and mindfulness (Ulrichsen et al., 2016). Another review found no significant effects for a broad range of methods, including traditional Chinese medicine, CBT, community health management, circuit training, hyperbaric oxygen therapy, music therapy, and respiratory training (Su et al., 2020). In conclusion, various treatment studies, both pharmacological and non-pharmacological, are being conducted, many without control groups.

In general, treatment options for fatigue are currently scarce in the healthcare, are requested by patients and need to be developed. However, a hindrance to developing treatment for fatigue is the absence of consensus defining fatigue after different illnesses as well as lack in knowledge about the origin of fatigue. Different terms for fatigue are used, for example, injury or illness-related definitions such as MS-fatigue or stroke-fatigue. Further, the terms, brain fog or cognitive fatigue are used with the intention to include cognitive difficulties within the concept of fatigue (Eng et al., 2019). In addition, central fatigue is used to emphasize that fatigue is based on dysfunction in the brain (Chaudhuri & Behan, 2004; Rönnbäck & Johansson, 2022). In this article, we focus on pathological mental fatigue with long-term consequences for patients. This is distinct from the fatigue that healthy people can experience, from which they recover after rest and sleep. More specifically, in this study, we focus on the mental and cognitive aspects of fatigue and use the Mental Fatigue Scale as an assessment of fatigue (MFS) (Johansson et al., 2010; Johansson & Rönnbäck, 2014a). The MFS include items on cognitive, sensory, and emotional difficulties which are consequences when endurance and mental energy are reduced, and fatigue will be perceived. These symptoms are all commonly reported after neurological disorders (Lindqvist & Malmgren, 1993) and the symptoms that are measured with MFS have been shown to be closely related with a high internal consistency (Johansson et al., 2010; Johansson & Rönnbäck, 2014a). In this study, we have chosen to use the term, brain fatigue (BF) with the intention to clarify the coexistence of fatigue and cognitive function as well as the central origin of this based on our hypothesis of a dysfunction in the astroglial support of glutamate transmission (Rönnbäck & Johansson, 2022). We define BF as a reduced capacity to continue a mental activity repeatedly and having a reduced ability to restore energy levels after mental activities (Johansson & Rönnbäck, 2014b). MFS is used as a subjective measure to indicate whether and to what extent BF is present.

We suggest that patients who suffer from BF need education and knowledge about its common symptoms. They need to learn and to adapt strategies and methods to alleviate BF and to find relief from the emotional burden associated with it. The psychological burden is considerable with emotional problems such as depression and/or anxiety, frustration, feelings of inadequacy, not being understood and not being able to take part in social life. A program for people suffering from BF combining these elements is lacking. Therefore, we present a new group program emanating from our previous experience of the program, Mindfulness Based Stress Reduction (MBSR) (Kabat-Zinn, 2001) used for participants suffering from BF (Johansson, Bjuhr, et al., 2012; Johansson, Bjuhr, et al., 2015). MBSR is a therapeutic program based on eastern meditative practices, interwoven with modern psychological and educational theories (Crane et al., 2017). MBSR was developed by Jon Kabat Zinn and colleagues back in 1979 and have introduced and adapted mindfulness to the western society. It is about training inner attitudes of presence with attention, compassion, without striving nor judging and with wisdom (Kabat-Zinn, 2001). MBSR is a program with clear guidelines as to the content and for the competence of the teacher (Crane, 2017; Crane et al., 2012). MBSR is an eight session-group program, including a whole day’s retreat in silence and home assignments with daily guided meditations and practices introducing mindfulness into daily life. During the eight group meetings, meditation and gentle yoga are practiced and reflective discussions are led by the teacher, all with the intention to create a better the understanding of human suffering by exploring the mind, discovering oneself with interest and gentleness and to learn about alternative ways of approaching life. The themes and discussions during the program are described as the ‘heart of the course’ (McCown et al., 2011).

The MBSR program has been suggested to reduce fatigue, depression, anxiety and improve cognition in illnesses affecting the brain, such as stroke, TBI and MS (Acabchuk et al., 2021; Azulay et al., 2013; Bédard et al., 2012; Grossman et al., 2004; Johansson, Bjuhr, et al., 2012; Johansson, Bjuhr, et al., 2015; Joo et al., 2010). In addition, meeting with and learning from others are described as being highly valuable, and can enhance learning and mindfulness practice (Cormack et al., 2018). From our previous experience with the MBSR program with patients suffering from BF after an acquired brain injury, some experienced the program as too intense and demanding with all the homework and daily meditation sessions each week (Johansson, Bjuhr, et al., 2012). In addition, MBSR is not adapted to patients suffering from BF and knowledge about BF is not explicitly informed on. When suffering from BF, we suggest that there are hindrances that need to be managed with the following strategies: a slower pace, repetition of information, shorter talks and discussions with intermediate breathing space, and more time between the meetings for home assignments and meditation sessions. The MBSR program includes long meditation sessions, with a duration of up to 45 minutes and this has been deemed acceptable for those suffering from BF (Johansson, Bjuhr, et al., 2012). A newly published book presents a similar approach based on the Mindfulness-based Cognitive Therapy (MBCT) program, also using long meditation sessions and biweekly sessions for participants suffering from chronic fatigue (McKechnie, 2023). In addition, the teacher or therapist needs to have a profound knowledge of BF as well as the teaching of the MBSR program.

The aim of this randomized pilot study is to evaluate a new Brain Fatigue and Mindfulness program (BF-M) for participants suffering from long-term BF after an acquired brain injury or the neurological disorder, MS. As BF is, to a large degree trans-diagnostic, is based on many years’ clinical experience, and since people who had suffered a TBI or stroke, or BF after MS report very similar symptoms on the MFS (Johansson et al., 2010), we chose to have groups with a mixed diagnosis. A mixed method research design is used including quantitative and qualitative methods. The quantitative study will compare a BF-M group with a waitlist control group. The primary outcome is the MFS score and the secondary outcome depression, anxiety, and quality of life. The research question guiding our qualitative analysis is: What experiences do patients with BF have of BF-M in the context of their BF? As this is a new program, a qualitative evaluation of BF-M can provide a clearer understanding of how BF-M works from the viewpoint of the participants.

## Method

### Participants

Participants with stroke, TBI, brain tumor, brain inflammation, MS and cardiac arrest were recruited via advertisement on the Swedish Neuro Association's website, from a Facebook group for people suffering from BF and from outpatient neuro- and rehabilitation clinics. For inclusion, interviews covered the personal history (anamnestic) and assessment of BF. The inclusion criteria were age between 18 and 65 years and having suffered from BF for at least three months. Exclusion criteria included severe psychiatric disorder, drug abuse and not being able to follow the instructions due to cognitive impairment. After inclusion, the participants were randomized to a BF-M group or a waitlist control group, using the excel random function. The waitlist participants were later offered the BF-M. They were then divided into two BF-M and two control groups as we wanted to keep the number of group participants to a maximum of 10, with the intention to reduce the impressions inflicted on the participants and to retain the sense of a calm space.

The study was approved by the Swedish Ethical Review Authority (2021-03125). All participants signed an informed consent for the study and were informed that they could withdraw at any time without giving any reason.

### BF-M

BF-M consists of six biweekly 2-hour meetings, with a duration of two hours each, over a total period of 11 weeks. At each meeting, meditations are practiced, and knowledge is imparted about BF. BF-M starts with gaining an understanding of BF and common symptoms. BF-M continues by exploring emotions and reactions in relation to BF and is followed by finding a wholesome balance in everyday life between rest and activity. During the meetings, group discussions are included with reflections around specific themes. This enables the participants to share experiences and to learn from each other. During the meetings, longer meditation sessions are practiced as well as shorter breath meditations. BF-M is conducted at a slower pace with repetition of information. BF-M is offered in a place with yoga mats, cushions and chairs and the participants can choose from these and can adapt to their own needs. The intention is to create an atmosphere of open-mindedness, compassion and acceptance. Participants receive pre-recorded meditations available on a website including 45-minute body scan, 20-minute sitting meditation, 45-minute sitting meditation, breathing space meditation, compassion meditation and mountain meditation and they are encouraged to practice daily meditations between the meetings. They also receive a video covering BF and common symptoms to be watched after session one and a video about strategies and how to manage BF to be watched after session four. In addition, they receive a booklet presenting the BF-M program and the themes for each session, which exercises to practice to next time and short texts about the key themes for each session. BF-M is presented in Table 1.

**Table 1.** Schematic presentation of the Brain Fatigue – Mindfulness program (BF-M).

Session	Meditations	Themes	
		BF	Mindfulness
1	Body scan 45 min.	Central and common symptoms	Awareness, Patience Non-judging, Non-striving, Curiosity
2	Body scan 45 min. Breathing space	Explore brain fatigue	Reactions and emotions
3	Sitting meditation 20 min. Breathing space	Explore emotions	Acceptance
4	Sitting meditation 45 min. Breathing space	Helpful strategies	To let go and letting be
5	Compassion meditation, 20 min. Breathing space	What is beneficial for me	Self-compassion
6	Mountain meditation, 20 min. Breathing space	The way forward	Generosity

### Quantitative assessment

Self-reported questionnaires were answered before the randomization and the start of BF-M and within one week after BF-M ended. The questionnaires included the MFS (Johansson & Rönnbäck, 2014a), Comprehensive Psychopathological Rating Scale (CPRS) for depression and anxiety (Svanborg & Åsberg, 1994), and the Brunnsviken Brief Quality of Life Scale (BBQ) (Lindner et al., 2016). The MFS has been evaluated for people with acquired brain injuries (Johansson et al., 2010; Johansson & Rönnbäck, 2014a). The patient needs to have had BF for at least one month, and a value above 10 indicates significant problem with BF. The MFS includes the following items: generalized fatigue, fatigue related to mental activities, mental recovery time, concentration and memory problems, slowness of thinking, stress sensitivity, emotional sensitivity (tearfulness) and irritability, reduced initiative, light, and sound sensitivity and sleep disturbances. The items have a high internal consistency (Cronbach’s alpha of 0.944) (Johansson & Rönnbäck, 2014a). The CPRS is used for depression and anxiety (Svanborg & Åsberg, 1994). Mild depression is indicated with a rating between 6.6 and 9.5, moderate between 10 and 17 and severe ≥ 17.5 (Snaith et al., 1986). The CPRS depression scale is identical to the Montgomery Åsberg Depression Rating Scale (MADRS) except that the rating is doubled in the MADRS (Montgomery & Åsberg, 1979). No cutoff value exists for anxiety. The BBQ measures importance-adjusted satisfaction across six life areas as leisure, view on life, creativity, learning, friends and friendship and view on self. The BBQ scale displays high concurrent and convergent validity and good reliability. The scale can differentiate between clinical and healthy groups and is sensitive to change (Lindner et al., 2016).

### Statistical analysis

Repeated analysis of variance for comparison between BF-M group and the wailist control group and post-hoc analysis with paired t-test were performed. For background data t-test or Chi-square test was used.

### Qualitative assessment

All 13 interviews with volunteers were conducted by the first author (GG) within a few weeks of having finished the BF-M. Four participants were interviewed in a calm room at the university hospital, four by phone and five by video call. The length of the interviews ranged between 20 and 62 minutes and lasted for 33 minutes on average. All interviews were conducted in Swedish and extracts presented have been translated into English. All quoted participants were assigned a number to ensure anonymity. Amongst the 13 participants who were interviewed, eleven suffered from ABIs and two from MS.

All participants took part in a structured interview comprising questions concerning how they experienced BF-M, whether and how they utilized information from BF-M, and other questions concerning BF-M in relation to BF. Follow-up questions were asked to encourage participants to elaborate on their answers. In total, the interview included sixteen questions, for example: What do you think of the program you have taken part in and what has it meant to you? What have you learned by participating in BF-M? What impact has the BF-M had on your daily life? Is there anything in BF-M that you have found particularly challenging? Is there anything in BF-M that you have found particularly positive?

### Qualitative analysis

Our analytic focus was on how participants experienced BF-M. For this purpose, we conducted an inductive semantic thematic analysis with a critical realist approach. That is, we viewed the participants’ responses as descriptors of their actual experiences, while at the same time realizing that their descriptions were subject to contextual influences. In our analyses, extracts could be coded according to multiple themes. We coded the interviews using ATLAS.ti (Version 23.0.1). Below is a specification on how the six phases of thematic analysis (Braun & Clarke, 2006) with revisions (Braun & Clarke, 2006, 2021) were conducted within this study:

1. First, GG created verbatim transcripts of all interviews. These were read and re-read by GG to familiarize himself with the material. In this phase, GG aimed to be open-minded about the material, even though the focus of the analysis was to serve the aim of the study.
2. In the second phase, GG generated initial codes from all interviews. In total, 101 codes were generated in this phase.
3. In the third phase, GG merged codes from the initial coding. This process did not imply passively searching for merging possible hits rather actively generating clusters of codes. In this phase, GG refrained from finalizing the clusters and instead he contemplated the different versions of it. At the end of this phase, the 101 codes had been merged into 49 codes of which four codes were based on at least 35 quotations each.
4. In the fourth phase, GG created a thematic structure based on the analysis in Phase 3 to test the fit of the themes in relation to both the code level of the data as well as the dataset as a whole. The main themes were created from three of the four clusters of codes with more than 35 quotations each that were generated in Phase three. This phase also involved reviewing whether the themes could be regarded as coherent and sound. After reviewing the thematic structure that was built on the three clusters of codes, GG opted to proceed with that structure.
5. The fifth phase comprised the process of naming and defining the themes.
6. In the final step, GG selected relevant extracts from the transcripts to exemplify the themes and to generate a descriptive account of how the themes corresponded with our aim. In this step, BJ and GG collaborated to review and revise the analysis.

There was a slight concern that GG’s and BJ’s pre-understanding of mindfulness treatments would influence the analysis. GG had taken part in a non-clinical MBSR program as a participant and BJ is a certified MBSR teacher with many years’ clinical experience working with BF patients in rehabilitation clinics and research experience. To separate the role of treatment designer and researcher, GG conducted and analyzed the interviews himself. He avoided reading the treatment material before interviewing the participants and analyzing the material. This served the purpose of collecting and analyzing the material without the direct influence of the terminology and rationale involved in the treatment. After GG had finalized a first version of the analysis, BJ and GG discussed the analysis and reflected upon the risk of bias in the analysis. The discussions involved verifying that the analysis was founded on the interviewees’ experiences and not directly on the BF-M material nor on the pre-understandings of GG and BJ. In general, we followed the suggestions of Levitt et al. (Levitt et al., 2018) concerning rigor and transparency in order to communicate our research in a comprehensible manner.

## Results

**Figure 1.**
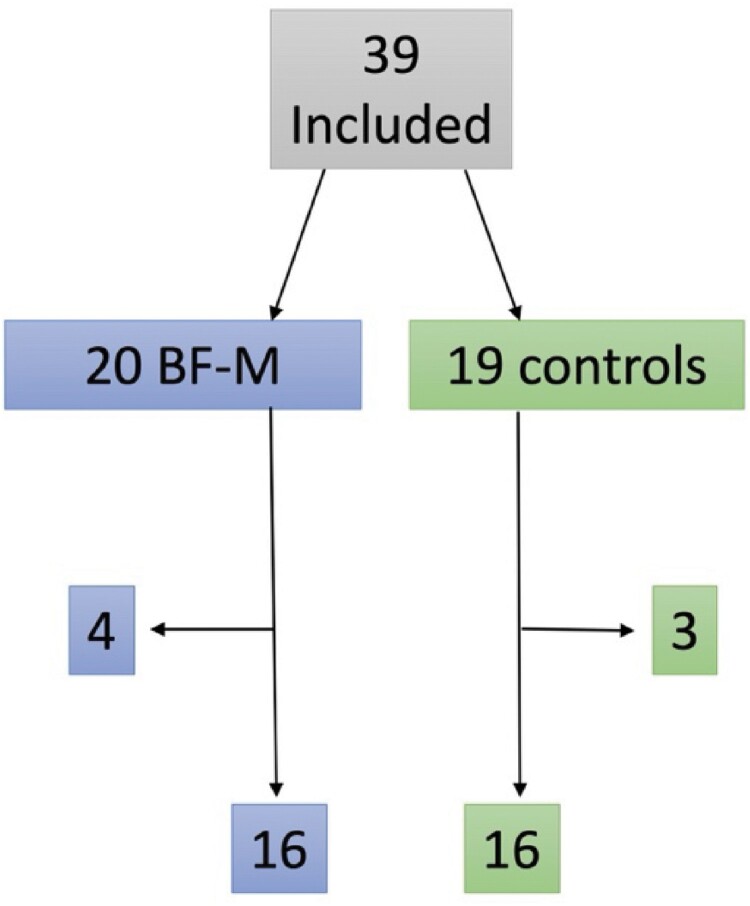
Flowchart of the study.

Thirty-nine participants with a mean age of 48 years were included and were randomized into two BF-M and two control groups. The first part (BF-M and control) was run in spring 2022 and the second in autumn 2022. Seventy-four percent were women and 26% men and the participants were evenly divided between the BF-M and control groups. The groups did not differ in age, sex, education, diagnosis, time since falling ill, working hours and rating on MFS (Table 2, 4). In total 16 participants that completed BF-M and 16 controls responded to the questionnaires before the randomization and the start of BF-M and within one week after BF-M ended. Four men dropped out of the BF-M, three of whom were working part – or full-time and dropped out after one session and one who had never started the treatment due to personal circumstances. Three of the controls only responded at baseline and, after having been sent reminders did not return the questionnaires in the pre-stamped envelopes (see flowchart in Figure 1). Thirteen of the controls have participated in BF-M (2 groups in total) later on.

**Table 2.** Background data of participants, age, gender, education, diagnosis, time since falling ill and working hours in relation to full-time work (100%).

	BF-M group	Control group	*p*-value
Age (years)	49.1 (9.6)	48.1 (11.6)	0.804
Range	33–64	29–64	
Females/males	12/4	12/4	1.000
Education: High school/university	5/11	9/7	0.154
Traumatic brain injury	3	6	
Stroke	5	3	
Brain inflammation	2	4	0.363
Brain tumor	1	0	
Cardiac arrest	0	1	
Multiple Sclerosis	5	2	

Note: Mean (sd), frequency and statistical comparison (*p*-value) between the groups with t-test or Chi-square.

### Quantitative analysis

The repeated ANOVA revealed a significant interaction between the groups with a large effect size for BF (MFS) and anxiety (CPRS) (Table 3). The post-hoc test (paired t-test) showed a significant reduction in MFS with a large effect size and a significant reduction with a medium effect size for anxiety (CPRS) for the BF-M group (Table 4). No significant change in depression (CPRS) and quality of life (BBQ) was detected in any of the groups and no significant change was detected for any of the variables in the control group (Table 4).

**Table 3.** A repeated two-factorial ANOVA comparing BF-M and control groups.

	Time	Interaction	Group
MFS^1^, mental fatigue	*F* = 11.936 *p* = 0.002 $\eta_{p}^{2}$ = 0.285	*F* = 6.757 *p* = 0.014 $\eta_{p}^{2}$ = 0.184	*F* = 3.379 *p* = 0.076 $\eta_{p}^{2}$ = 0.101
CPRS^2^, depression	*F* = 1.375 *p* = 0.250 $\eta_{p}^{2}$ = 0.045	*F* = 0.065 *p* = 0.801 $\eta_{p}^{2}$ = 0.002	*F* = 0.407 *p* = 0.529 $\eta_{p}^{2}$ = 0.014
CPRS^2^, anxiety	*F* = 2.170 *p* = 0.152 $\eta_{p}^{2}$ = 0.070	*F* = 5.511 *p* = 0.026 $\eta_{p}^{2}$ = 0.160	*F* = 0.535 *p* = 0.470 $\eta_{p}^{2}$ = 0.018
BBQ^3^, quality of life	*F* = 0.947 *p* = 0.339 $\eta_{p}^{2}$ = 0.033	*F* = 0.161 *p* = 0.692 $\eta_{p}^{2}$ = 0.006	*F* = 1.635 *p* = 0.212 $\eta_{p}^{2}$ = 0.055

Note: *F*-value, *p*-value, and effect size (partial eta square, $\eta_{p}^{2}$). ^1^Mental Fatigue Scale. ^2^Comprehensive Psychopathological Rating Scale. ^3^Brunnsviken Brief Quality of Life Scale. Effect size; partial eta square: 0.01 = small, 0.06 = medium, 0.14 = large.

**Table 4.** Post-hoc test, paired t-test for each group separately.

	BF-M		Controls	
	mean (sd)	*p*-value (d)	mean (sd)	*p*-value (d)
Mental fatigue, MFS	21.0 (4.3)^1^ 18.1 (5.7)^2^	0.004 (0.843)	22.5 (3.5)^1^ 22.1 (3.8)^2^	0.347 (0.242)
Depression, CPRS	8.1 (2.7)^1^ 7.6 (2.9)^2^	0.292 (0.282)	8.7 (3.2)^1^ 8.3 (2.8)^2^	0.550 (0.153)
Anxiety, CPRS	9.2 (2.5)^1^ 7.8 (2.7)^2^	0.037 (0.596)	7.8 (2.2)^1^ 8.1 (2.5)^2^	0.462 (0.189)
Quality of life, BBQ	50.8 (20.7)^1^ 54.4 (23.2)^2^	0.439 (0.206)	43.9 (15.0)^1^ 45.4 (12.9)^2^	0.581 (0.146)

Note: Mean (sd) at baseline/start^1^ and 12 weeks after BF-M had finished^2^, *p*-value and the effect size Cohen’s d. Effect size; Cohen’s d: 0.2 = small, 0.5 = medium, 0.8 = large.

### Qualitative analysis

The aim of the qualitative analysis in this study was to explore participants’ experiences of the BF-M. To address this aim, we conducted and analyzed interviews with 13 participants in relation to our research question: What experiences do patients with BF have of BF-M in the context of their BF? By analyzing the interviews, we generated three themes with eight sub-themes that jointly answered our research question. These, along with example quotes for each sub-theme are presented in Table 5. In our analysis, themes serve as descriptors for related sub-themes (Robinson, 2011) while sub-themes illustrate our analysis of specific experiences that the participants described.

**Table 5.** Overview of themes, sub-themes and example quotes.

Theme	Subtheme	Example Quote
A Social Space for People Suffering from BF	Sharing Experiences	‘Just this to get together in a group and then share some experiences’ (IP5)
	Feeling More Understood	‘Finally, I meet a person who understands without me having to explain and that feeling is irreplaceable’ (IP4)
	Feeling less Alone	‘So, you need to talk to each other, you need to juggle (experiences), that's what you need and just hear or see that person, so you don't feel alone with your problems, that's kind of the thing with this like, that's the therapeutic thing with these kinds of courses’ (IP3)
Understanding and Living with BF	Learning about one’s Condition and Associated Reactions	‘I have tried to resist not being sick or ill, and it is clear that it somehow takes a lot of energy when you should really try to understand that you are not like you were before’ (IP13)
	Learning about one’s Needs	Well, I think I also landed a bit more in the fact that I’m brain tired, I’ve been brain tired for a long time […] and that i need to rest (IP8)
	Gaining Acceptance	‘That's what I thought was good about this acceptance, that it becomes like a different approach too, so the other participants in the course, they had probably all been sick longer than me and some will never get well, so accept the situation as it is […] So I think and hope that it will be like normal again, on the other hand it's more acceptance if it doesn't turn out that way, and it can be good anyway so I (laughs) well I think it’s going to be fine’ (IP10)
A Toolbox for Managing BF	Applied Tools	‘The different forms and the different exercises, it created a certain flexibility based on what the different days look like and what method you can use for each day. Sometimes you might want a longer meditation lying down and sometimes you might have to take a shorter one at the workplace […] and it's good to have that toolbox I think’ (IP7)
	Strategic Tools	‘I think I have gained a greater insight as I need to find space for mindfulness exercises because I feel that it has a positive effect and that's probably what has been most important that you have gained that insight and that from that you can build a strategy to make it work in everyday life’ (IP7)

#### Theme 1. A social space for people suffering from BF

This theme describes the participants’ experiences that BF-M facilitated a social meeting space for people suffering from BF. The BF-M became a space for the participants to exchange experiences associated with BF. Thus, this theme addressed our aim by suggesting that the BF-M met this need among the participants for exchange in relation to BF-associated experiences. In our analysis, there were three functions that this social space fulfilled: (1) Sharing Experiences, (2) Feeling More Understood, and (3) Feeling less Alone. In the following, we describe these subthemes in greater detail.

*Sharing Experiences.* Sharing experiences of BF with others affected by the condition was a prominent aspect of the perceived value of BF-M. Sharing experiences of BF had to do with the exchange of issues associated with BF but also the sharing of tips and tricks that participants had learned in relation to living with BF. In our analysis, it became apparent that many of the participants had little or no previous experience of being in a situation or a space together with other people affected by BF. BF-M served the purpose of facilitating this opportunity.

*Feeling More Understood.* In our analysis, the sharing of experiences implied the opportunity to feel more understood. For many participants this seemed to be the first time they had found themselves in a situation where other people genuinely understood their challenges. Participants commonly shared the experience that colleagues, family members and even healthcare professionals in general had very limited knowledge of BF. Thus, BF-M became an opportunity to share this experience of feeling misunderstood but was also a space where they could feel genuinely understood by the other participants.

*Feeling Less Alone*. Closely related to the feeling of being understood were participants' experiences of feeling less alone which they had gained by participating in the BF-M. Having previously had limited or no contact with other people suffering from BF, participants described the experience of being able to share their struggles with others and they thus felt less alone. As suggested in the example quote for this sub-theme (Table 1), the feeling of not being alone with one’s problem can have a therapeutic effect and can imply empowerment as one continues to deal with BF.

#### Theme 2. Understanding and living with BF

This theme describes participants´ experiences of gaining insights into BF through BF-M. In line with the psycho-educative purpose of BF-M, participants shared experiences of how they had come to gain a clearer understanding of BF and the consequences of the condition. They experienced that: (1) they had learned about their condition and associated reactions, (2) they had learned about their needs, and (3) they had gained some acceptance concerning the limitations BF implies. Next, we describe these sub-themes in further detail.

*Learning about one’s Condition and associated Reactions.* This sub-theme describes how participants experienced that BF-M meant an opportunity to learn about their condition and associated reactions. By learning from the BF-M material, by taking part in the exercises and by sharing experiences with other participants, they experienced that they had gained insights into BF and the consequences of the condition. This implied that participants became aware of their symptoms and how these affected their functionality in everyday life. From these insights, participants could learn to anticipate their needs in different situations. As suggested in the example quote (Table 5), resisting the challenge of acknowledging one’s condition could be a strain. According to our analysis, learning about one’s condition and about one’s reactions to it was a necessary means with which to move forward and meet the challenges presented by BF.

*Learning about one’s Needs.* By gaining a clearer understanding of their condition, participants described that they became more attentive to their needs. This was clearly communicated by participants, as illustrated in the example quote (Table 5). For example, this could involve avoiding the lunchroom at work during busy hours, taking a break from family time when feeling the need to, or avoiding paying bills in the end of a working day. Taking all the impressions gained from the participants´ descriptions, BF-M meant that they had learned to become more attentive to their needs that stem from BF. That said, putting this understanding into practice could be challenging as participants experienced that their responsibilities as employees and householders were not always compatible with attending to their BF-related needs.

*Gaining Acceptance.* As illustrated in the example quotes (Table 5), meeting others and learning about the BF conditions, could create the opportunity to learn to accept the limitations involved. While several participants pointed out this opportunity, they also seemed to have hope in becoming better at this. Balancing acceptance and hope, as suggested in the example quote (Table 1), was one way to deal with the risk of having to live with chronic BF. Indeed, participants shared the experience of having met with other participants who had lived with BF for a long period of time and realizing that they themselves could also be affected by BF in the long-term. This was not only a stark realization for them but it was also an opportunity to learn to accept their condition and to work with it. In our analysis, gaining acceptance is a psychological process rather than an event. Striking a balance between acceptance and hope illustrates this process.

#### Theme 3. A toolbox for managing BF

This theme was generated to illustrate that participants experienced that BF-M provided them with the tools for dealing with BF. Our analysis of their experiences suggests that the tools provided served two purposes for the participants. Firstly, they gained (1) tools for use in everyday life as the day or week unfolded. Secondly, they gained (2) strategic tools that allowed them to plan and to formulate strategies to cope with BF over time. Next, we describe these sub-themes in greater detail, below:

*Applied Tools.* Participants shared their experiences of how BF-M had provided them with tools for use in everyday life. For example, short breath meditations were used by many participants throughout the day to manage their BF and reduce the risk of exhaustion. Participants described having practiced a five-minute breath meditation amid their working day, either in the car before grocery shopping or during time spent with their family. Participants also used longer mediations as an applied tool in their everyday lives. For example, one participant reported regularly practicing a 45-minute meditation in-between work and family time. In our analysis, participants´ descriptions of the different tools suggested that these were applied throughout the day and throughout the week to help them to cope with BF. The flexibility associated with a multifaceted set of tools is clearly communicated in the example quote (Table 5).

*Strategic Tools.* In addition to applied tools, our analysis of the participants’ experiences also suggested that BF-M provided them with strategic tools. This is illustrated in the example quote (Table 5). Strategic tools allowed participants to plan and formulate a strategy for managing their everyday lives so that they could maintain good functionality and, at the same time taking care of their BF. Thus, strategic tools implied that participants planned their lives in such a way that they did not become exhausted. For example, when interviewed, some participants pointed this out by explaining that they had planned for a free schedule after the interview so that they had time to rest and recover. Other participants described having formulated long-term strategies to manage a balance between work and family. These kinds of short- and long-term strategies were used to sustain a manageable everyday life.

## Discussion

This pilot study is an initial evaluation of a new program, BF-M, designed for participants suffering from long-term BF after an acquired brain injury or neurological disorder, such as MS. The results showed a significant reduction in BF (MFS) and anxiety (CPRS), and the interviews emanated in the following three overarching themes; (1) BF-M became a space for the participants to exchange and share experiences associated with BF, (2) the participants felt more understood and less alone and (3) gaining a clearer understanding of living with BF. The participants reported having learned about their condition, reactions and needs and having gained a greater acceptance of the condition. BF-M also gave them strategies to manage and cope with BF in everyday life using meditations and applying strategies to fit their needs. This all shows promising results for BF-M.

All participants had suffered from long-term BF, most of them for several years. They were all of working age, a few were working part-time or full-time but most were not able to work due to their BF. This is in accordance with previous reports, showing the impact of BF on work ability (Andersen et al., 2012; Balasooriya-Smeekens et al., 2016; Johansson, 2022; Norlander et al., 2021; Rutkowski et al., 2021). Not being able to manage work was frustrating, but it was also frustrating not to be able to manage daily living including family and friends. This was a common subject during the discussions and meetings. From the interviews, we learned that BF-M has provided a social space well-suited for people suffering from BF. The participants could share experiences, feel more understood and less alone since all of them were suffering from BF. This opportunity to share experiences with peers who had a pre-understanding of one’s condition seemed to be important and confirms previous research findings concerning the benefits of practicing in a group (O'Dowd & Griffith, 2022). According to our analysis, BF-M allowed the participants to share experiences and to better understand and live with their condition. These results correspond well with previous research findings that have highlighted the group-learning effect of mindfulness-based interventions (Kvillemo & Bränström, 2011; Malpass et al., 2015; Visser et al., 2015). Our analysis added new knowledge by yielding specific insights on how the participants had come to better understand and live with BF. This was achieved partly by learning about the needs associated with BF specifically and partly by gaining acceptance of their BF which could empower them to manage the condition. In addition, our study has revealed specific applied and strategic tools that the participants had come to use through having taken part in BF-M. Similarly, the use of coping techniques through mindfulness programs has been shown in other patient groups (Bogosian et al., 2016; Malpass et al., 2015; Müller-Engelmann et al., 2017).

Furthermore, the participants had different diagnoses and the groups were mixed. It could be considered favorable not to mix diagnoses; in that way the patients had more in common with each other. However, from previous studies with BF using MFS, similar BF symptoms are reported among acquired brain injuries and MS (Johansson et al., 2010), and as BF is the theme in BF-M, a mixture of diagnoses may be less significant and this is in accordance with the results from the interviews. From a qualitative study with participants from mindfulness-based interventions, it was concluded that when a teacher builds a safe and supportive group environment the participants are able to benefit from their shared experiences (Cormack et al., 2018), and this also correlates with the findings in our study.

Our results from this initial pilot study on BF-M suggest that patients suffering from BF need education and knowledge about the common symptoms related to fatigue. Due to diminished cognitive functions, they need to learn and adapt strategies to alleviate BF and find relief from the resultant emotional burden. From previous treatment studies, reduction of BF is reported, but none of the studies have included a psycho-educative program specifically designed for BF. From our previous research experience, it was clear that patients need to learn about BF, to learn useful strategies and to adapt to their personal capabilities, as the energy budget remains limited even with methylphenidate.

From the knowledge we have today, BF needs to be prioritized and treatment options need to be implemented in health care, these being tailored to suit the individual patient’s needs (Eng et al., 2019; McKevitt et al., 2010). Jointly, the results from our mixed-methods design have provided evidence that BF-M may be an option for further evaluation as a treatment option for BF. The randomized quantitative study suggested that BF-M may have a clinically significant effect on reducing BF. The analyses of the qualitative interviews provided insights into what this effect might imply from the patient’s perspective.

### Limitations

This is the first study on BF-M and the number of participants is low. The intention was to understand the usefulness of BF-M in relation to BF. Such information needs to be generated as a first step in assessing whether to further invest in the evaluation of a treatment. Studies with greater numbers of participants and other patient-groups are warranted. With larger groups, it could be interesting to compare effects for different diagnostic groups. This study was too small for that, but the rating on MFS between the MS (mean 20.5) and acquired brain injury (mean 22.1) did not differ in this study. We chose a waitlist control group design since it is difficult to design an adequate control group situation not having ascertained a potential effect on BF. Several factors can have an effect on BF, such as relaxing and being calm for a while, cognitive behavioral therapy (Nguyen et al., 2017), group meetings, education concerning BF and learning strategies for coping with BF. For this initial pilot study on BF-M, we only wanted to evaluate the feasibility and whether it was possible to reduce the BF. The intention was not to compare different treatments. For this purpose, a waitlist control group was regarded as the best option.

The recruitment of participants was not evenly divided between men and women. More women (74%) were represented. The same problem with gender imbalance is reported from MBSR studies with women representing 2/3 (Bodenlos et al., 2017). More research is warranted to explore this, and modifications may be needed to better attract men with BF. Unfortunately, we do not have any comments from those who dropped out since, according to the ethical rules, participants could cancel without providing any comments. The reasons for not continuing can only be speculative. Concerning the interviews, studies with follow-up interviews conducted later on, rather than in adjacent to BF-M, could yield varying results due to differences in population characteristics, practice frequency and other time-related factors. However, in this study the interviews were carried out in conjunction with BF-M, thereby ameliorating memory challenges that commonly affect patients with acquired brain injuries. This precautionary approach has been suggested when interviewing patients with acquired brain injuries (Niraj et al., 2018).

## Conclusion

In conclusion, we have designed a program combining meditation sessions with psycho-educative group discussions and imparting knowledge about BF. This new BF-M program for participants suffering from BF after acquired brain injury or MS reduced BF (MFS) and anxiety. Sharing experiences associated with BF was important as well as feeling more understood and feeling less alone. Participants had learned about their condition and how to better cope with BF. Our results suggest that BF-M may be a rehabilitation option for BF after acquired brain injuries, neurological illnesses and possibly for other disorders where BF is a common and long-lasting condition. BF patients seek treatment as an essential aspect of healthcare, with a growing demand for evidence-based approaches in addressing their needs. Since this pilot study on BF-M suggests it has promise as a treatment for the relief of the symptoms of BF amongst different patient groups, we encourage further evaluation of the feasibility of BF-M with larger randomized studies.

## Data Availability

Data can be shared upon reasonable request.
